# Suicide ideation and psychotropic recreational drug use by adolescents: a systematic review and meta-analysis

**DOI:** 10.1590/1516-3180.2022.0641.R2.23012024

**Published:** 2024-04-22

**Authors:** Cássia Lima de Oliveira Gracini, Gustavo Giacomelli Nascimento, Maria Tereza Campos Vidigal, Murilo Navarro de Oliveira, Álex Moreira Herval, Cauane Blumenberg, Walbert A. Vieira, Rafael Rodrigues Lima, Luiz Renato Paranhos

**Affiliations:** I MSc. Nurse, Master’s student, Postgraduate Program in Management and Public Health, Universidade Estadual de Campinas (UNICAMP), Piracicaba (SP), Brazil.; II PhD. Dentist, Principal Investigator, National Dental Centre Singapore, National Dental Research Institute Singapore, Singapore, Singapore; Professor, Oral Health Academic Clinical Programme, Duke-NUS Medical School, Singapore, Singapore.; III MSc. Dentist, Master’s student, Postgraduate Program in Dentistry, School of Dentistry, Universidade Federal de Uberlândia (UFU), Uberlândia (MG), Brazil.; IV MSc. Dentist, Doctoral student, Postgraduate Program in Dentistry, School of Dentistry, Universidade Federal de Uberlândia (UFU), Uberlândia (MG), Brazil.; V PhD. Dentist, Professor, Division of Preventive and Community Dentistry, School of Dentistry, Universidade Federal de Uberlândia (UFU), Uberlândia (MG), Brazil.; VI PhD. Computer Scientist, Collaborative Researcher, Postgraduate Program in Epidemiology, Federal University of Pelotas, Pelotas, Brazil.; VII MSc. Dentist, Doctoral student, Department of Restorative Dentistry, Endodontics Division, School of Dentistry of Piracicaba, Universidade Estadual de Campinas (UNICAMP), Piracicaba (SP), Brazil.; VIII PhD. Dentist, Professor, Laboratory of Functional and Structural Biology, Institute of Biological Sciences, Universidade Federal do Pará, Belém (PA), Brazil.; IX PhD. Dentist, Professor, Division of Preventive and Community Dentistry, School of Dentistry, Federal University of Uberlândia, Uberlândia, Brazil.

**Keywords:** Adolescent, Psychotropic Drugs, Systematic review, Suicide, Substance-related disorders, Drug use, Similar predictors, Suicide ideation

## Abstract

**BACKGROUND:**

Adolescence is characterized by complex and dynamic changes, often involving experimentation, including the use of psychotropic substances. Although it is well-established that recreational psychotropic drugs are associated with suicide ideation in adults, evidence of this association in adolescents remains limited.

**OBJECTIVE:**

To investigate the relationship between suicide ideation and psychotropic recreational drug use among adolescents.

**DESIGN AND SETTING:**

Systematic review with meta-analysis developed at Universidade Federal de Uberlândia (UFU) and Universidade Estadual de Campinas (UNICAMP), Brazil.

**METHODS:**

A search across eight electronic databases for observational studies, without language or publication year restrictions, was conducted. The Joanna Briggs Institute tool was used to assess the risk of bias. Random-effects meta-analyses and odds ratios were used to measure the effects.

**RESULTS:**

The search yielded 19,732 studies, of which 78 were included in the qualitative synthesis and 32 in the meta-analysis. The findings indicated that suicidal ideation was 1.96 times more likely (95% confidence interval, CI = 1.47; 2.61) for adolescents who used some drug recurrently and 3.32 times more likely (95%CI = 1.86; 5.93) among those who abused drugs. Additionally, adolescents who used cannabis were 1.57 times more likely (95%CI = 1.34; 1.84) to experience suicide ideation compared with non-users, while cocaine users had 2.57 times higher odds (95%CI = 1.47; 4.50).

**CONCLUSIONS:**

Psychotropic recreational drug use is associated with suicidal ideation among adolescents regardless of current or previous use, abuse, or type of substance used.

**SYSTEMATIC REVIEW REGISTRATION:**

Registered in the PROSPERO database under the identification number CRD42021232360. https://www.crd.york.ac.uk/prospero/display_record.php?ID=CRD42021232360.

## INTRODUCTION

Adolescence marks a period characterized by complex and dynamic changes that directly influence individuals’ personalities and social performance development.^1^ These transformations contribute to the development of various types of behaviors, including experimentation with drugs,^2^ alcohol, medications, and other psychoactive substances,^3^ making adolescence a critical phase vulnerable to starting substance use, including psychotropic drugs.^4^ Drug use disorders severely affect children and adolescents’ physical and mental health.^5^

The scientific literature underscores the widespread prevalence of psychotropic substance use among adolescents globally. A study performed in Poland showed that 10.8% of respondents aged 13 to 17 had consumed psychotropic drugs, such as cannabis, cocaine, or heroin, at least once.^6^ Similarly, a survey on drug use among Brazilian adolescents indicated cannabis as the second most commonly used substance, following alcohol.^7^

Different factors contribute to adolescents’ susceptibility to drug consumption.^7-10^ In the family environment, permissive relationships regarding smoking and alcohol consumption, particularly among males, predict substance use.^8^ Furthermore, social determinants such as strain family relationships,^7^ low maternal education levels, non-white-collar parental occupations, economic hardships within the community, and high poverty and unemployment levels at 18 years are the risk factors for drug use in adolescence.^10^ In the school environment, adolescents who experience or perpetrate bullying demonstrate a higher prevalence of substance use.^9^

A study by the United Nations Office on Drugs and Crime revealed a 30% global increase in drug use between 2009 and 2018,^11^ along with a 10% increase in suicide rates between 2006 and 2018.^12^ Annually, 800,000 individuals worldwide die by suicide, making it the second principal cause of death among young women and the third among young men.^13^

Suicidal ideation in adolescents is influenced by biological, psychological,^14^ and socioeconomic factors.^15^ Developing suicidal ideation, meaning the existence of suicidal thoughts by adolescents, is related to the feeling of not belonging to the school environment, low resilience, the existence of stress factors throughout life,^16^ the presence of depressive symptoms, bullying and other types of violence, and tobacco and alcohol consumption.^17,18^ Symptoms such as sadness, self-hatred, fatigue, self-deprecation, and crying are associated with depression and suicidal ideation, with loneliness being particularly correlated.^19^ The prevalence of suicidal ideation among adolescents ranges between 15 and 25%, representing a significant global public health care concern.^20^

A previous systematic review of 108 included studies highlighted positive associations between different substances, such as alcohol, tobacco, cannabis, illicit drugs, and non-medical use of prescription drugs and suicidal ideation in adults.^21^ However, robust evidence evaluating the consumption of psychotropic recreational drugs and their relationship with suicidal ideation among adolescents remains limited.

## OBJECTIVE

This systematic review aimed to compare suicidal ideation among adolescents who did and did not use psychotropic recreational drugs.

## METHOD

### Protocol registration

The protocol for this systematic review was based on the Preferred Reporting Items for Systematic Review and Meta-Analysis Protocols (PRISMA-P) guidelines^22^ and registered in the International Prospective Register of Systematic Reviews (PROSPERO) database (http://www.crd.york.ac.uk/PROSPERO) under the number CRD42021232360. The Preferred Reporting Items for Systematic Reviews and Meta-Analyses (PRISMA) guidelines^23^ and the Joanna Briggs Institute (JBI) Manual for Evidence Synthesis^24^ were used to conduct this systematic review.

### Research question

This systematic review aimed to answer the following guiding question based on the Population, Exposition, Comparator, and Outcome (PECO) acronym: “Is the use of psychotropic recreational drugs associated with a higher chance of suicide ideation among adolescents?”

### Inclusion criteria

Observational studies (prospective or retrospective) comparing suicidal ideation (outcome) between adolescents in the school environment (population) who reported psychotropic recreational drug use (exposition) and adolescents who did not use psychotropic recreational drugs (comparator).

Including the classifications of the World Health Organization (10–19 years old) and the United Nations (15–24 years old), the age range of 10–24 years was considered as adolescents in this study.

We defined cannabis, cocaine, ecstasy, LSD, heroin, amphetamine, glue-sniffing, and cracks as psychotropic drugs. The assessment of suicidal ideation can be primary (questionnaire/interview) or secondary (database or reports).

There were no restrictions on publication language or year.

### Exclusion criteria

The exclusion criteria were as follows: studies that evaluated the relationship between alcohol or tobacco use alone and suicide ideation, studies performed in psychiatric clinics, studies with specific groups of adolescents (ethnic minorities, indigenous populations, or adolescents with mental disorders), studies that did not include adolescents in the school environment, studies performed with university students, or postmortem assessments.

### Sources of information and search

Electronic searches were performed in the MedLine (*via* PubMed), Scopus, LILACS, SciELO, Embase, and Web of Science databases. OpenThesis and OpenGrey were used to capture the “gray literature” partially. The MeSH (Medical Subject Headings), DeCS (Health Sciences Descriptors), and Emtree (Embase Subject Headings) were used to select the search descriptors. Synonyms and free words were used in the search. The Boolean operators “AND” and “OR” were used to improve the research strategy with several combinations. Table 1 lists the details of the combinations used in each database. A bibliographic search was conducted for articles published until January 2022. The results obtained in the primary databases were initially exported to the EndNote Web™ software (Thomson Reuters, Toronto, Canada) for cataloging and removing duplicates. The other results were exported to Microsoft Word (Microsoft™, Ltd, Washington, United States) for manually removing duplicates.

**Table 1:** Strategies for database search

Databases	Search Strategy
Main databases	
**PubMed** http://www.ncbi.nlm.nih.gov/pubmed	((( "Psychotropic Drugs" OR "Psychoactive Agents" OR "Psychoactive Drugs" OR "Substance Use" OR "Narcotic" OR "Substance-Related Disorders" OR "Substance Abuse" OR "Drug Abuse") AND ("Suicide" OR "Suicides" OR "Suicidal" OR "Self-harm" OR "Self-Injurious Behavior" OR "Self Destructive Behavior" OR "Suicidal Ideation" OR "Self-Destructive Behavior" OR "Attempted Suicide") AND ("Adolescence" OR "Adolescent" OR "Student" OR "Teen" OR "Teenager" OR "Youth" OR "Young" )))
**Embase** https://www.embase.com	(("Psychotropic Drugs" OR "Psychoactive Agents" OR "Psychoactive Drugs" OR "Substance Use" OR "Narcotic" OR "Substance-Related Disorders" OR "Substance Abuse" OR "Drug Abuse") AND ("Suicide" OR "Suicides" OR "Suicidal" OR "Self-harm" OR "Self-Injurious Behavior" OR "Self Destructive Behavior" OR "Suicidal Ideation" OR "Self-Destructive Behavior" OR "Attempted Suicide") AND ("Adolescence" OR "Adolescent" OR "Student" OR "School" OR "Teen" OR "Teenager" OR "Youth" OR "Young"))
**Web of Science** http://apps.webofknowledge.com/	(((("Psychotropic Drugs" OR "Psychoactive Agents" OR "Psychoactive Drugs" OR "Substance Use" OR "Narcotic" OR "Substance-Related Disorders" OR "Substance Abuse" OR "Drug Abuse") AND ("Suicide" OR "Suicides" OR "Suicidal" OR "Self-harm" OR "Self-Injurious Behavior" OR "Self Destructive Behavior" OR "Suicidal Ideation" OR "Self-Destructive Behavior" OR "Attempted Suicide") AND ("Adolescence" OR "Adolescent" OR "Student" OR "School" OR "Teen" OR "Teenager" OR "Youth" OR "Young"))))
**SciELO** http://www.scielo.org/	(("Psychotropic" OR "Psychoactive Agents") AND ("Suicide" OR "Suicidal") AND ("Adolescent" OR "Teenager" OR "Youth")) (("Drug Abuse" OR "Substance Abuse" OR "Narcotic") AND ("Suicide" OR "Suicidal") AND ("Adolescence" OR "Teen" OR "Young"))
**LILACS** http://lilacs.bvsalud.org/	(("Psychotropic" OR "Psychoactive Agents") AND ("Suicide" OR "Suicidal") AND ("Adolescent" OR "Teenager" OR "Youth")) AND (instance:" regional") AND ( db:( "LILACS")) tw:((("Drug Abuse" OR "Substance Abuse" OR "Narcotic") AND ("Suicide" OR "Suicidal") AND ("Adolescent" OR "Teenager" OR "Youth" ))) AND (instance:" regional") AND ( db:( "LILACS"))
**Scopus** http://www.scopus.com	(("Psychotropic Drugs" OR "Psychoactive Agents" OR "Psychoactive Drugs") OR ("Suicide" OR "Suicides" OR "Suicidal" OR "Self-harm") OR ("Adolescence" OR "Adolescent" OR "Student" OR "School" OR "Teen" OR "Teenager" OR "Youth" OR "Young")) (("Narcotic" OR "Substance-Related Disorders" OR "Substance Abuse" OR "Drug Abuse") OR ("Self-Injurious Behavior" OR "Self Destructive Behavior" OR "Suicidal Ideation" OR "Self-Destructive Behavior" OR "Attempted Suicide") OR ("Adolescence" OR "Adolescent" OR "Teen" OR "Teenager"))
**Gray literature**	
**OpenThesis** https://oatd.org	(("Substance Use") AND ("Suicide") AND ("Adolescent"))
**OpenGrey** http://www.opengrey.eu/	(("Substance Use" OR "Drug Abuse" OR "Substance Abuse") AND ("Suicide" OR "Suicidal" OR "Self-harm" OR "Self-Injurious Behavior" OR "Self-Destructive Behavior") AND ("Adolescent" OR "Teenager" OR "Adolescence" OR "Teen" OR "Young"))

### Study selection

Before study selection, a calibration exercise was performed. The authors discussed the eligibility criteria and applied them to a sample of 20% of the retrieved studies to determine the inter-examiner agreement. After reaching a proper level of agreement (Kappa ≥ 0.81), the reviewers performed a methodical analysis of the titles of the studies (first phase), eliminating those not pertinent to the topic. In the second phase, the same reviewers evaluated the abstracts of the studies using the initial eligibility criteria. Titles that met the study objectives but lacking available abstracts were analyzed in the next phase. In the third phase, reviewers read the full texts of the eligible studies to confirm adherence to the eligibility criteria. Excluded studies in this phase were registered separately, accompanied by explanations for exclusion. In cases where full texts were unavailable, requests were made to library databases for bibliographic assistance, and e-mails were sent to the corresponding authors to obtain the texts. Two reviewers independently performed all phases; in cases of doubt or disagreement, a third reviewer was consulted to make the final decision.

### Data collection

Before data extraction, to ensure consistency between the reviewers, a calibration exercise was performed in which data from three eligible studies were extracted together by a third reviewer. Subsequently, the two eligible reviewers extracted the following information from the studies: identification of the study (author, year, country, and study location), sample characteristics ( number of patients in each study, nationality, sex, and average age), collection and processing characteristics (type of questionnaire and/or interview applied, drugs used by adolescents), and the main results ( presence of suicide ideation in user and non-user adolescents, odds ratio – OR). In case of incomplete or insufficient information, the corresponding author was contacted via email.

### Risk of bias assessment

The JBI Critical Appraisal Checklist for Analytical Cross-Sectional Studies was used to analyze the risk of bias and individual methodological quality of the studies selected.^25^ Two reviewers independently assessed each domain regarding the potential risk of bias, as recommended by the PRISMA statement.^23^ Each study was categorized according to the rate of positive answers to the questions corresponding to the assessment tool. The risk of bias was considered high when the study obtained 49% of answers as “yes”, moderate when the study obtained 50% to 69% of answers as “yes”, and low when the study reached more than 70% of “yes” answers.^26^

### Data synthesis and Meta-analysis

Meta-analyses were conducted to aggregate the primary findings of the eligible studies and compare the OR of suicide ideation between the exposed (drug-user adolescents) and non-exposed (non-drug-user adolescents) groups. A separate meta-analysis was conducted foreach type of substance (e.g., cannabis, cocaine, and psychotropic drug use) and user profile (e.g., dependence, current use, and use at some point in life) if at least three studies provided sufficient and comparable information. When included studies provided multiple OR estimates, the model with the highest number of adjusted variables was selected for inclusion. For longitudinal studies, the OR from the first follow-up wave was selected.

Heterogeneity among the studies was measured using three indicators: the I^2^, indicating the rate of variability caused by heterogeneity among studies; H^2^, denoting the level of heterogeneity (H = 1 indicating homogeneity); and τ^2^, representing the variance among studies. All analyses were performed with random effects, considering that the high heterogeneity estimates observed in the meta-analysis models.

Funnel plots were produced to verify the publication bias in the different meta-analyses, but only for the models that included 10 or more studies.^27^ Additionally, sensitivity tests were conducted, including only studies with a low risk of bias, to assess the impact of the individual risk of bias of the eligible studies in the meta-analysis. All statistical analyses were performed using the Stata 16.1 software (StataCorp LLC, College Station, TX, USA), with asignificance level set at 5 %.

### Certainty of evidence

The certainty of evidence was assessed using the Grading of Recommendation, Assessment, Development, and Evaluation (GRADE) tool. The GRADE pro-GDT software (http://gdt.guidelinedevelopment.org) was used to summarize the results. The assessment was based on study design, risk of bias, inconsistency, indirect evidence, imprecision, and publication bias. The certainty of evidence can be classified as high, moderate, low, or very low.^28^

## RESULTS

### Study selection

**Figure 1:**
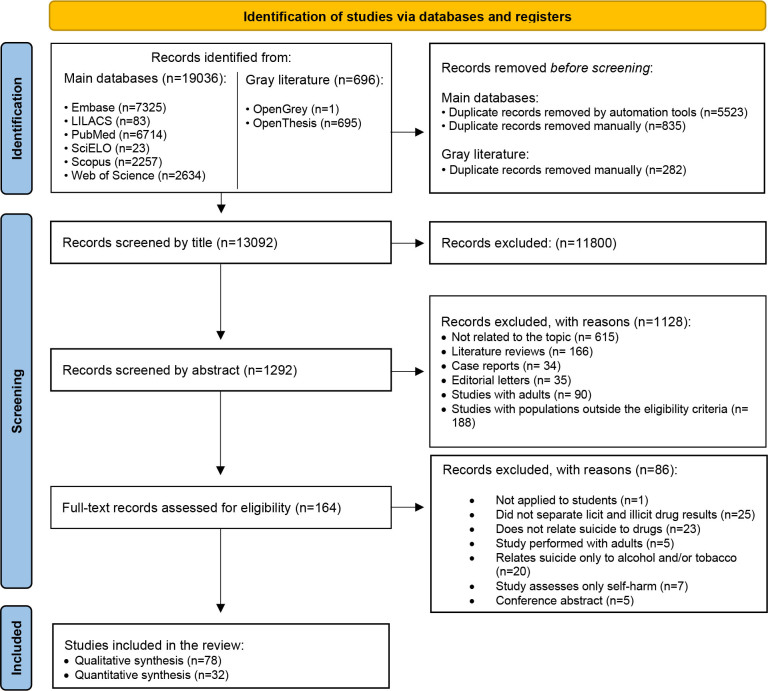
Flowchart depicting the study selection process (Preferred Reporting Items for Systematic Reviews and Meta-Analyses flow diagram).

During In the initial phase of study selection, 19,732 hits were identified. Following the removal of duplicates, 13,092 results underwent screening based in the title and abstract. Subsequently, 164 studies met the eligibility criteria and underwent full-text analysis. Among these, 78^17,18,29-104^ were included in the qualitative synthesis of results (Figure 1). Supplementary Table 1 provides details regarding the exclusion of 86 studies.

### Study characteristics

The selected studies were published between 1991 and 2020, with 38 studies performed in North America, 12 in Asia, 11 in Europe, eight in Africa, four in South America, four in Oceania, and one intercontinental study in the USA and France. Regarding data collection methods, 56 studies collected their data from secondary databases, while 22 utilized questionnaires. Among the Cannabis was the most frequently studied drug in relation to suicide ideation, followed by cocaine, inhalants, injectable drugs, ecstasy, methamphetamine, glue, heroin, and crack. Among studies reporting the number of research participants, the total sample sizewas 1,122,111 answers, with 51.63% women and 48.36% men. Table 2 and Table 3 detail the main characteristics and outcomes of the eligible studies, respectively.

**Table 2:** Main characteristics of the eligible studies

Author, year^ref^	Country	Age (years)	Average age	n	Data source
Kandel et al., 1991^29^	USA	NR	NR	593 (NR♂;NR♀)	Self-administered questionnaire
Felts et al., 1992^30^	USA	NR	NR	3,064 (NR♂;NR♀)	North Carolina Youth Risk Behavior Survey (1990)
Garrison et al., 1993^31^	USA	NR	NR	3,674 (1,702♂;2,062♀)	South Carolina Youth Risk Behavior Survey (1990)
Vega et al., 1993^32^	USA	NR	NR	5,303 (NR♂;NR♀>)	Self-administered questionnaire
Madianos et al., 1994^33^	Greece	12-17	NR	4,291(1,940♂;2,351♀)	Self-administered questionnaire
Burge et al., 1995^34^	USA	NR	NR	11,631 (5,676♂;5,955♀)	1990 Youth Risk Behavior Survey (USA)
Lopez et al., 1995^35^	Mexico	13-19	NR	3,459 (1,764♂;1,695♀)	National High-School Survey – Mexico 1992
Windle and Windle, 1997^36^	USA	n.r.	15.54 ± 0.66	975 (458♂;517♀)	Self-administered questionnaire
Simon and Crosby, 2000^37^	USA	NR	NR	16,296 (NR♂;NR♀)	1993 National School-Based Youth Risk Behavior Survey (YRBS)
Perkins and Hartless, 2002^38^	USA	12-17	NR	14,922 (NR♂;NR♀)	Self-administered questionnaire
Vermeiren et al., 2003^39^	Belgium	12-18	14.9 ± 1.9	794 (794♂;0♀)	Self-administered questionnaire
Hallfors et al., 2004^40^	USA	15-19	NR	18,922 (9,288♂;9,634♀)	Wave-I in-home contractual data set of Add Health (1994)
Wu et al., 2004^41^	USA	9-17	NR	1,458 (NR♂;NR♀)	NIMH Methods for the Epidemiology of Child and Adolescent Mental Disorders (MECA) Study and The Westchester Study
Yip et al., 2004^42^	China	15-24	15.8	2,586 (306♂;42♀)	Hong Kong Youth Sexuality Survey - 2001
Spremo and Loga, 2005^43^	Bosnia-Herzegovina	16-18	NR	202 (51♂;151♀)	Self-administered questionnaire
Ulusoy et al., 2005^44^	Turkey	17-18	NR	726 (306♂;420♀)	Self-administered questionnaire
Chabrol et al., 2008^46^	France	15-20	16.7 ± 1.3 (♂) 17 ± 1.3 (♀)	248 (76♂;172♀)	Self-administered questionnaire
Dunn et al., 2008^45^	USA	NR	12.9 ± 2.64 (♂) 12.8 ± 2.61 (♀)	10,273 (5,126♂;5,146♀	Self-administered questionnaire
Luncheon et al., 2008^47^	USA	NR	NR	7,544 (0♂;7,544♀)	2003 Youth Risk Behavioral Surveillance System
Peltzer et al., 2008^48^	South Africa	NR	15.78 ± 1.58	1,157 (358♂;79♀)	Self-administered questionnaire
Epstein and Spirito, 2009^49^	USA	NR	NR	10,273 (5,126♂;5,146♀)	Youth Risk Behavior with probability proportional to school enroll Surveillance (United States, 2005)
Peter and Roberts, 2010^50^	Canada	15	NR	2,499 (1,222♂;1,277♀)	National Longitudinal Survey of Children and Youth – Waves 3 to 6
Pickles et al., 2009^51^	England	NR	NR	2,226 (NR♂>;NR♀	Baseline of the Isle of Wight study, an epidemiological sample of adolescents assessed in 1968
Florenzano et al., 2010^52^	Chile	NR	NR	2,322 (1,026♂;1,296♀)	Self-administered questionnaire
Page et al., 2011^53^	China and Philippines	11-17	NR	16,353 (7,450♂;8,725♀)	Global School Health Survey (2003) – Data from China and Philippines
Souza et al., 2010^54^	Brazil	11-15	NR	1,039 (501♂;538♀)	Self-administered questionnaire
Wolitzky-Taylor et al., 2010^55^	USA	12-17	NR	7,637 (NR♂;NR♀)	National Survey of Adolescents (1995/2005)
Alwan et al., 2011^56^	Seychelles	11-17	14 ± 1.4	1,432 (NR♂;NR♀)	Self-administered questionnaire
Carvalho et al. 2011^57^	Brazil	14-19	NR	4,201 (1,688♂;2,513♀)	Self-administered questionnaire
Eaton et al., 2011^58^	USA	NR	NR	6,322 (0♂;6,322♀)	Youth Risk Behavior Survey (2007)
Kim et al., 2011^59^	USA	12-17	NR	19,301 (NR♂;NR♀)	2000 National Household Survey on Drug Abuse (NHSDA)
Miller et al., 2011^60^	Mexico	12-17	NR	3,005 (NR♂;NR♀)	Mexican Adolescent Mental Health Survey (MAMHS)
Swahn et al., 2011^61^	USA and France	11-19	NR	28,323 (13,895♂;14,398♀)	2003 European School Survey Project on Alcohol and Other Drugs (France) and 2003 Youth Risk Behavior Survey (USA)
Ahmad et al., 2012^62^	Malaysia	12-17	NR	25,507 (12,498♂;13,009♀)	2012 Malaysia Global School-based Student Health Survey
Bakken and Gunter, 2012^63^	USA	NR	NR	2,548 (1,274♂;1,274♀)	Delaware High School Youth Risk Behavior Survey (YRBS-H)
Kokkevi et al., 2012^64^	17 European countries	15-16	NR	45,806 (NR♂;NR♀)	European School Survey Project on Alcohol and Other Drugs (ESPAD) - 2007
Peltzer and Pengpid, 2012^65^	Thailand	12-15	NR	2,758 (1,364♂;1,394♀)	Thailand Global School-Based Health Survey (2008)
Wilson et al., 2012^66^	Seychelles	11-17	NR	1,432 (687♂;745♀)	Global School-based Student Health Survey – Data from Seychelles
Arenliu et al., 2013^67^	Kosovo	15-16	15.65 ± 0.68 (♂) 15.63 ± 0.69 (♀)	4,709 (2,112♂;2,597♀)	2011 European School Survey Project on Alcohol and other Drugs (ESPAD)
Consoli et al. 2013^68^	France	17	NR	36,757 (18,164♂;18,593♀)	Self-administered questionnaire
Delfabbro et al., 2013^69^	Australia	14-16	15.2 ± 0.5	2,552 (1,041♂;1,485♀)	Self-administered questionnaire
Govender et al., 2013^70^	South Africa	13-17	14.7 ± 0.74	239 (112♂;127♀)	Self-administered questionnaire
Rasic et al., 2013^71^	Canada	NR	NR	976 (486♂;490♀)	Self-administered questionnaire
Shilubane et al., 2013^72^	South Africa	13-19	NR	20,646 (9,878♂;10,768♀)	2002 and 2008 South African Youth Risk Behaviour Surveys
Van Ours et al., 2013^74^	New Zealand	10-24	NR	938 (459♂;479♀)	Christchurch Health and Development Study (CHDS)
Wong et al., 2013^73^	USA	NR	NR	73,183 (37,104♂;36,079♀)	Data from the 2001 to 2009 Youth Risk Behavior Survey
Chabrol et al., 2014^75^	France	NR	17.1 ± 1.2 (♂) 16.7 ± 1 (♀)	972 (594♂;378♀)	Self-administered questionnaire
Lowry et al., 2014^76^	USA	NR	NR	14,000 (NR♂;NR♀)	11 national Youth Risk Behavior Surveys conducted biennially between 1991 and 2011
Randall et al., 2014^77^	Benin	12-16	NR	2,690 (NR♂;NR♀)	Global School-based Health Survey (2009) – Data from Benin
Zhang and Wu, 2014^78^	USA	11-21	NR	3,342 (NR♂;NR♀)	Public-use Add Health – Wave 1
Delfabbro et al., 2015^79^	Australia	14-16	15.2 ± 0.5	2,552 (1,041♂;1,485♀)	Self-administered questionnaire
Dunlavy et al., 2015^80^	Tanzania	11-16	NR	2,154 (1,034♂;1,120♀)	Global School-Based Student Health Survey (2006) – Data from Dar el Salaam
Gart and Kelly, 2015^81^	USA	NR	16 ± 1.2	15,363 (7,655♂;7,708♀)	2011 Youth Risk Behavior Survey
Lee and Choi, 2015^82^	South Korea	13-18	NR	72,435 (35,655♂;35,780♀)	2013 Online Survey of Youth Health Behavior in Korea
Peltzer and Pengpid, 2015^17^	4 countries of Oceania	13-16	NR	6,540 (2,846♂;3,534♀)	Global School-Based Health Survey (2011) – Data from Samoa, Kiribati, Salomon Island, and Vanuatu
Sampasa-Kanyinga et al., 2015^83^	Canada	11-20	14.4 ± 1.9	1,922 (883♂;3,534♀)	Ontario Student Drug Use and Health Survey (2009/2011/2013)
Sharma et al., 2015^84^	Peru	12-18	NR	916 (425♂;491♀)	Self-administered questionnaire
Dudovitz et al., 2015^85^	EUA	NR	NR	15,698 (7,656♂;8,042♀)	2011 Youth Risk Behaviors Survey
Ammerman et al., 2016^86^	USA	NR	NR	4,834 (2,315♂;2,419♀)	Longitudinal Study of Adolescent Health (2009)
DeCamp and Bakken, 2016^87^	USA	NR	NR	4,834 (2,315♂;2,419♀)	2005, 2007, and 2009 Delaware High School Youth Risk Behavior Survey (YRBS-H)
Price and Khubchandani, 2016^88^	USA	NR	NR	13,721 (NR♂;NR♀)	Youth Risk Behavior Survey (2001/03)
Weeks and Colman, 2016^90^	Canada	12-17	NR	6,788 (3,287♂;3,501♀)	National Longitudinal Survey of Children and Youth
Agrawal et al., 2017^91^	USA	12-22	NR	3,277 (NR♂;NR♀)	Baseline of the Collaborative Study of the Genetics of Alcoholism
Asante et al., 2017^92^	Ghana	NR	NR	1,973 (1,065♂;908♀	Ghana Global School-based Student Health Survey (2012)
Janssen et al., 2017^93^	France	17	NR	22,023 (11,034♂;10,989♀)	Self-administered questionnaire
Wang and Yen, 2017^94^	Taiwan	12-19	14.75 ± 1.77	13,985 (NR♂;NR♀)	2004 Project for the Health of Adolescents in Southern Taiwan
El Kazdouh et al., 2018^95^	Morocco	14-19	NR	800 (374♂;426♀)	Self-administered questionnaire
Haskuka et al., 2018^96^	18 European countries	15	NR	105,000 (NR♂;NR♀)	European School Survey Project on Alcohol and Other Drugs (ESPAD – 2011)
Subica and Wu, 2018^97^	USA	12-18	NR	184,494 (NR♂;NR♀)	1991 – 2015 Combined National Youth Behavioral Risk Surveys
Chadi et al., 2019^98^	USA	NR	NR	26,821 (13,062♂;13,749♀)	Two waves (2015 and 2017) of the national Youth Risk Behavior Survey
Dema et al., 2019^99^	Bhutan	13-17	NR	5,809 (2,554♂;3,255♀)	Global School-Based Student Health Survey (2016) – Data from Bhutan
Georgiades et al., 2019^100^	Canada	14-17	NR	2,396 (1,189♂;1,207♀)	2014 Ontario Child Health Study
Jung et al., 2019^101^	South Korea	NR	NR	59,984 (30,384♂;29,600♀)	Korea Youth Risk Behavior Web-based Survey (2017)
Baiden et al., 2020^18^	USA	NR	NR	13,697 (6,609♂;7,088♀)	2017 Youth Risk Behavior Survey (YRBS)
Greene et al., 2020^102^	USA	NR	NR	16,390 (8,149♂;8,187♀)	2013 New Mexico Youth Risk and Resiliency Survey (NM-YRRS)
Khan et al., 2020^103^	Bangladesh	11-18	NR	2,989 (1,952♂;1,037♀)	Global School-based Student Health Survey (2014) – Data from Bangladesh
Kim et al., 2020^89^	South Korea	12-17	15.9 ± 0.02	65,528 (33,803♂;31,725♀)	2016 Korea Youth Risk Behavior Web-based Survey (KYRBS)
Sakamoto et al., 2020^104^	USA	14-18	NR	1,943 (982♂;951♀)	Youth Risk Behavior Survey (2017) – Data from the Northern Mariana Islands

♂= Male ♀= Female; NR = Not reported.

**Table 3:** Qualitative synthesis of the main results of the eligible studies

Author^ref^	Main outcomes
**Kandel et al.** ^29^	There was a strong association between psychotropic recreational drug use and suicidal ideation in female adolescents.
**Felts et al.** ^30^	Drug use, particularly crack and cocaine, was associated with suicidal ideation.
**Garrison et al.** ^31^	Illicit psychotropic recreational drug use was significantly associated with suicidal ideation.
**Vega et al.** ^32^	Illicit psychotropic recreational drug use was consistently related to higher levels of suicidal ideation.
**Madianos et al.** ^33^	The severity and frequency of substance use influenced the prevalence of suicidal ideation in the sample analyzed.
**Burge et al.** ^34^	Psychotropic recreational substance use showed a positive association with suicidal ideation.
**Lopez et al.** ^35^	Psychotropic recreational drug use represented a risk factor for suicidal ideation among the students.
**Windle and Windle** ^36^	Psychotropic recreational drug use did not show a significant relationship with suicidal ideation, but it did with suicide attempts.
**Simon and Crosby** ^37^	There was a relationship between psychotropic recreational substance use and suicidal ideation and thoughts.
**Perkins and Hartless** ^38^	There was a relevant association between the use of hard psychotropic recreational drugs and suicidal ideation.
**Vermeiren et al.** ^39^	Psychotropic recreational substance use did not show a significant association with suicidal ideation.
**Hallfors et al.** ^40^	Psychotropic recreational drug use, particularly injectable drugs, presented a significant association with suicidal ideation.
**Wu et al.** ^41^	The association between psychotropic recreational substance use and abuse was not significant after controlling adolescent depression.
**Yip et al.** ^42^	Illicit psychotropic recreational drug use was not considered an important risk factor for suicidal ideation.
**Spremo and Loga** ^43^	Psychoactive substance use presented an important connection to the presence of suicidal ideation in the adolescents studied.
**Ulusoy et al.** ^44^	There was no significant association between illicit psychotropic recreational drug use and suicidal ideation, but adolescents who smoke cigarettes were more prone to ideation.
**Chabrol et al.** ^46^	There were significant associations between psychotropic recreational substance use and suicidal ideation among adolescents attending rural schools.
**Dunn et al.** ^45^	Cannabis use by adolescents was significantly associated with suicidal behavior (including suicidal ideation).
**Luncheon et al.** ^47^	Illicit psychotropic recreational drugs were seriously associated with suicidal ideation and thoughts.
**Peltzer et al.** ^48^	The involvement with psychotropic recreational drugs was not associated with suicide risk (including suicidal ideation).
**Epstein and Spirito** ^49^	Sniffing glue had no significant relationship with suicidal ideation, but injecting drugs showed a high association with suicidal ideation.
**Peter and Roberts** ^50^	Cannabis use did not show a significant relationship with suicidal ideation.
**Pickles et al.** ^51^	Problems with psychotropic recreational substance use were considered risk factors for the increase in suicidal behavior.
**Florenzano et al.** ^52^	There was a high correlation between psychotropic recreational substance use and suicidal behavior (including suicidal ideation) and depression.
**Page et al.** ^53^	Psychotropic recreational drug use is significantly associated with higher levels of suicidal ideation.
**Souza et al.** ^54^	Illicit psychotropic recreational drug use was one of the factors related to suicidal ideation among adolescents.
**Wolitzky-Taylor et al.** ^55^	Psychotropic recreational substance use was significantly associated with an increased risk of suicidal ideation in adolescents in both years of the study.
**Alwan et al.** ^56^	Psychotropic recreational substance use was strongly associated with suicidal ideation among adolescents.
**Carvalho et al.** ^57^	Psychotropic recreational drug use was directly associated with suicidal ideation and planning among adolescents.
**Eaton et al.** ^58^	There was a significant association between psychotropic recreational drug use and suicidal ideation among adolescents.
**Kim et al.** ^59^	The use of ecstasy and other psychotropic recreational drugs presented a significant association with suicidal ideation.
**Miller et al.** ^60^	There was an association between psychotropic recreational substance use and suicidal ideation, and this relationship started with adolescents aged 13 years and increased according to age.
**Swahn et al.** ^61^	The early drug initiation of adolescents showed an association with suicidal ideation in both countries analyzed.
**Ahmad et al.** ^62^	The use of hard psychotropic recreational drugs was significantly related to suicidal ideation.
**Bakken and Gunter** ^63^	Psychotropic recreational substance use showed a significant relationship with suicidal behavior and ideation among the adolescents participating in the study.
**Kokkevi et al.^64>^**	Psychotropic recreational substance use was associated with suicidal ideation.
**Peltzer and Pengpid.** ^65^	Psychotropic recreational substance use did not show a strong association with suicidal ideation.
**Wilson et al.** ^66^	There was a strong association between suicidal ideation and illicit psychotropic recreational substance use for men.
**Arenliu et al.** ^67^	Substance use was associated with higher levels of suicide risk (including suicidal ideation), without differences between the sexes.
**Consoli et al.** ^68^	Psychotropic recreational substance use by adolescents was potentially related to higher rates of suicidal ideation and attempts.
**Delfabbro et al.** ^69^	Psychotropic recreational substance abuse was positively correlated with suicidal ideation.
**Govender et al.** ^70^	Illicit psychotropic recreational drug use was significantly associated with high levels of suicidal ideation among the adolescents in the study.
**Rasic et al.** ^71^	Psychotropic recreational substance use by the adolescents analyzed showed a significant association with suicidal ideation.
**Shilubane et al.** ^72^	Psychotropic recreational substance abuse was a strong risk factor for suicidal ideation and behavior, and this association increases with specific illicit drugs and the concomitant use of several substances.
**Van Ours et al.** ^74^	Intensive cannabis use led to higher levels of suicidal ideation in men.
**Wong et al.** ^73^	The levels of suicidal ideation were significantly higher among female adolescents with mental health problems and psychotropic recreational substance use and abuse.
**Chabrol et al.** ^75^	Cannabis use was not a significant independent predictor of suicidal ideation after adjusting the confounding factors of the total sample and subsample of cannabis users.
**Lowry et al.** ^76^	Psychotropic recreational substance use was significantly associated with the increased risk of suicide (including suicidal ideation) among the students.
**Randall et al.** ^77^	There was an association between illicit psychotropic recreational drug use and suicidal ideation.
**Zhang and Wu** ^78^	Psychotropic recreational drug use did not increase the risk of suicidal ideation, but suicidal ideation increased the risk of psychotropic recreational drug use.
**Delfabbro et al.** ^79^	Cannabis use did not show a significant association with suicidal ideation, but it was related to suicidal planning by adolescents.
**Dunlavy et al.** ^80^	Illicit psychotropic recreational drug use was associated with suicidal ideation and planning.
**Gart and Kelly** ^81^	Cannabis and cocaine use showed a significant relationship with suicidal ideation.
**Lee and Choi** ^82^	The use of psychoactive recreational drugs was associated with suicidal ideation among the different sexes and age groups of adolescents.
**Peltzer and Pengpid** ^17^	Psychotropic recreational substance use was one of the factors associated with suicidal ideation for the adolescents studied.
**Sampasa-Kanyinga et al.** ^83^	Adolescents who reported early psychotropic recreational drug use were more likely to report suicidal ideation.
**Sharma et al.** ^84^	Cannabis use was positively associated with suicidal ideation.
**Dudovitz et al.** ^85^	Illicit psychotropic recreational substance use did not show a significant association with suicidal ideation.
**Ammerman et al.** ^86^	Psychotropic recreational substance use showed a higher association with suicidal ideation than other risk behaviors.
**DeCamp and Bakken** ^87^	Psychotropic recreational substance use (cannabis and hard drugs) is significantly correlated with suicidal ideation among female heterosexual adolescents.
**Price and Khubchandani** ^88^	Psychotropic recreational substance abuse was associated with high levels of suicidal ideation.
**Weeks and Colman** ^90^	There was a positive relationship between psychotropic recreational substance use and suicidal ideation.
**Agrawal et al.** ^91^	Psychotropic recreational substance use by adolescents without a history of depression increases the risk of suicidal ideation.
**Asante et al.** ^92^	Early psychotropic recreational substance use by adolescents was not associated with suicidal ideation.
**Janssen et al.** ^93^	Psychotropic recreational substance use was not considered a risk factor for suicidal ideation.
**Wang and Yen** ^94^	The regular use of psychoactive substances presented a significant association with suicidal behavior (including suicidal ideation). This relationship was measured based on the mental health of adolescents.
**El Kazdouh et al.** ^95^	Psychotropic recreational substance use by adolescents was significantly associated with suicidal ideation, without differences between the sexes.
**Haskuka et al.** ^96^	Psychotropic recreational substance use was significantly associated with suicidal ideation for male adolescents, and this relationship increased with age.
**Subica and Wu** ^97^	Cannabis use presented a relationship with suicidal ideation in three of the 13 countries participating in the study.
**Chadi et al.** ^98^	Cannabis use showed a relationship with suicidal ideation only for some of the ethnic groups studied.
**Dema et al., 2019** ^99^	Marijuana use was associated with a higher likelihood of suicidal ideation and depressive symptoms.
**Georgiades et al.** ^100^	Psychotropic recreational drug abuse and the impulse to consume drugs were considered risk factors for suicidal ideation.
**Jung et al.** ^101^	The use of cannabis and other illicit substances did not show a significant association with suicidal ideation, but it did with suicide attempts.
**Baiden et al.** ^18^	Psychotropic recreational substance use was one of the factors associated with suicidal ideation.
**Greene et al.** ^102^	Illicit psychotropic recreational substance use was one of the factors associated with suicidal ideation, as well as a history of sexual abuse, bullying, and tobacco consumption.
**Khan et al.** ^103^	Psychotropic recreational substance use showed a significant association with suicidal ideation for both sexes.
**Kim et al.** ^89^	Adolescents who used psychotropic recreational drugs were more likely to present suicidal behavior (including suicidal ideation).
**Sakamoto et al.** ^104^	The use of hard psychotropic recreational drugs by male adolescents was associated with suicidal ideation.

### Assessment of the risk of bias of studies

Among the studies, 12 were deemed to have a moderate risk of bias, while 66 had a low risk; none were classified as having a high risk of bias. Question 1, regarding the eligibility criteria for sample selection, was answered negatively in nine studies. This answer is important because it favors sample standardization and decreases the risk of bias. Questions 5 and 6 were answered negatively in 11 studies, indicating that most studies identified confounding factors and established strategies for addressing them (Table 4).

**Table 4:** Risk of bias assessed according to the Joanna Briggs Institute (JBI) Critical Appraisal Tools for use in JBI Critical Appraisal Checklist for Analytical Cross-Sectional Studies

Author^ref^	Q1	Q2	Q3	Q4	Q5	Q6	Q7	Q8	% Yes	Risk
Kandel et al.^29^	--	√	--	√	√	√	--	√	62,5	Moderate
Felts et al.^30^	√	√	√	√	--	--	√	√	75	Moderate
Garrison et al.^31^	√	√	√	√	√	√	√	√	100	Low
Vega et al.^32^	√	√	√	√	--	--	√	√	75	Moderate
Madianos et al.^33^	√	√	√	√	√	√	--	√	87,5	Low
Burge et al.^34^	√	√	√	√	--	--	√	√	75	Moderate
Lopez et al.^35^	√	v	--	√	√	√	√	√	87,5	Low
Windle and Windle^36^	√	√	√	√	--	--	√	√	75	Moderate
Simon and Crosby^37^	√	√	√	√	√	√	√	√	100	Low
Perkins and Hartless^38^	--	√	√	√	√	√	√	√	87,5	Low
Vermeiren et al.^39^	√	√	√	√	√	√	√	√	100	Low
Hallfors et al.^40^	√	--	√	√	√	√	√	√	87,5	Low
Wu et al.^41^	√	√	√	√	√	√	√	√	100	Low
Yip et al.^42^	√	√	√	√	√	√	√	√	100	Low
Spremo and Loga^43^	--	√	√	√	--	--	√	√	62,5	Moderate
Ulusoy et al.^44^	√	√	√	√	--	--	√	√	75	Moderate
Chabrol et al.^46^	--	√	√	√	√	√	√	√	87,5	Low
Dunn et al.^45^	--	√	√	√	--	--	√	√	62,5	Moderate
Luncheon et al.^47^	√	√	√	√	√	√	√	√	100	Low
Peltzer et al.^48^	√	√	√	√	--	--	√	√	75	Moderate
Epstein and Spirito^49^	√	√	√	√	√	√	√	√	100	Low
Peter and Roberts^50^	√	√	√	√	√	√	√	√	100	Low
Pickles et al.^51^	√	√	√	√	√	√	√	√	100	Low
Florenzano et al.^52^	--	√	√	√	--	--	√	√	62,5	Moderate
Page et al.^53^	√	√	√	√	√	√	√	√	100	Low
Souza et al.^54^	√	√	√	√	√	√	√	√	100	Low
Wolitzky-Taylor et al.^55^	√	√	√	√	√	√	√	√	100	Low
Alwan et al.^56^	√	√	√	√	√	√	√	√	100	Low
Carvalho et al.^57^	√	√	√	√	√	√	√	√	100	Low
Eaton et al.^58^	√	√	√	√	√	√	√	√	100	Low
Kim et al.^59^	--	√	√	√	√	√	√	√	87,5	Low
Miller et al.^60^	√	√	√	√	√	√	√	√	100	Low
Swahn et al.^61^	√	√	√	√	√	√	√	√	100	Low
Ahmad et al.^62^	√	√	√	√	√	√	--	√	87,5	Low
Bakken and Gunter^63^	√	√	√	√	√	√	--	√	87,5	Low
Kokkevi et al.^64^	√	√	√	√	√	√	√	√	100	Low
Peltzer and Pengpid^65^	√	√	√	√	√	√	√	√	100	Low
Wilson et al.^66^	√	√	√	√	√	√	√	√	100	Low
Arenliu et al.^67^	√	√	√	√	√	√	√	√	100	Low
Consoli et al.^68^	√	√	√	√	--	--	√	√	75	Moderate
Delfabbro et al.^69^	√	√	√	√	√	√	√	√	100	Low
Govender et al.^70^	--	√	√	√	√	√	√	√	87,5	Low
Rasic et al.^71^	√	√	√	√	√	√	√	√	100	Low
Shilubane et al.^72^	√	√	√	√	√	√	√	√	100	Low
Van Ours et al.^74^	√	√	√	√	√	√	√	√	100	Low
Wong et al.^73^	√	√	√	√	√	√	√	√	100	Low
Chabrol et al.^75^	--	√	√	√	√	√	√	√	87,5	Low
Lowry et al.^76^	√	√	√	√	√	√	√	√	100	Low
Randall et al.^77^	√	√	√	√	√	√	√	√	100	Low
Zhang and Wu^78^	√	√	√	√	√	√	√	√	100	Low
Delfabbro et al.^79^	√	√	√	√	√	√	√	√	100	Low
Dunlavy et al.^80^	√	√	√	√	√	√	√	√	100	Low
Gart and Kelly^81^	√	√	√	√	√	√	√	√	100	Low
Lee and Choi^82^	√	√	√	√	√	√	√	√	100	Low
Peltzer and Pengpid^17^	√	√	√	√	√	√	--	√	87,5	Low
Sampasa-Kanyinga et al.^83^	√	√	√	√	√	√	√	√	100	Low
Sharma et al.^84^	√	√	√	√	√	√	√	√	100	Low
Dudovitz et al.^85^	√	√	√	√	√	√	√	√	100	Low
Ammerman et al.^86^	√	√	√	√	√	√	√	√	100	Low
DeCamp and Bakken^87^	√	√	√	√	--	--	√	√	75	Moderate
Price and Khubchandani^88^	√	√	√	√	√	√	√	√	100	Low
Weeks and Colman^90^	√	√	√	√	√	√	√	√	100	Low
Agrawal et al.^91^	√	√	√	√	√	√	√	√	100	Low
Asante et al.^92^	√	√	√	√	√	√	√	√	100	Low
Janssen et al^93^	√	√	√	√	√	√	√	√	100	Low
Wang and Yen^94^	√	√	√	√	√	√	√	√	100	Low
El Kazdouh et al.^95^	√	√	√	√	√	√	√	√	100	Low
Haskuka et al.^96^	√	√	√	√	√	√	√	√	100	Low
Subica and Wu^97^	√	√	√	√	√	√	√	√	100	Low
Chadi et al.^98^	√	√	√	√	√	√	√	√	100	Low
Dema et al., 2019^99^	√	√	√	√	√	√	√	√	100	Low
Georgiades et al.^100^	√	√	√	√	√	√	√	√	100	Low
Jung et al.^101^	√	√	√	√	√	√	√	√	100	Low
Baiden et al.^18^	√	√	√	√	√	√	√	√	100	Low
Greene et al.^102^	√	√	√	√	√	√	√	√	100	Low
Khan et al.^103^	√	√	√	√	√	√	√	√	100	Low
Kim et al.^89^	√	√	√	√	√	√	√	√	100	Low
Sakamoto et al.^104^	√	√	√	√	√	√	√	√	100	Low

Q1 = Were the inclusion criteria in the sample clearly defined?; Q2 = Were the study subjects and the setting described in detail?; Q3 = Was exposure measured validly and reliably?; Q4 = Were objective and standard criteria used for measuring the condition?; Q5 = Were confounding factors identified?; Q6 = Were strategies to deal with confounding factors stated?; Q7 = Were the outcomes measured validly and reliably?; Q8 = Was appropriate statistical analysis used?; √ = Yes; -- = No; NA = Not Applicable; U = Unclear.

### Meta-analysis

Although eligible studies analyzed the likelihood of suicide ideation relative to various types of drugs, only three comparisons were suitable for meta-analysis: 1) any psychotropic recreational drug use, 2) cannabis use, and 3) cocaine use. For any psychotropic recreational drug use, the analysis included three subtypes: adolescents who used it at some point in life, those who reported currently using the drug, and those who suffered from drug dependence or abuse. Regarding cannabis, only current users were analyzed, while regarding cocaine, the analysis was performed on adolescents who used the drug at some point in their lives.

### The use of any psychotropic recreational drug

**Figure 2:**
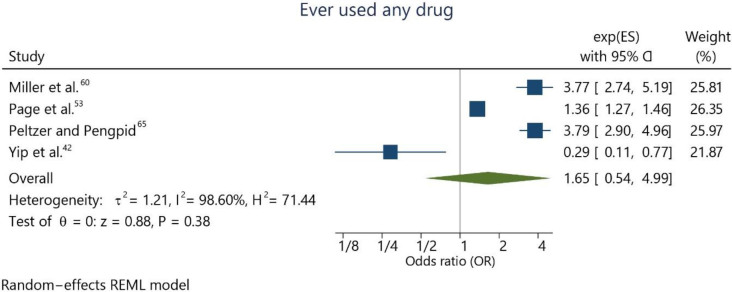
Meta-analysis assessing the use of any illicit drug at some point in life and suicide ideation.

Four studies^42,53,60,65^ evaluated the psychotropic recreational drug use at certain points in life. The combined effect estimation was OR 1.65 (95% confidence interval, CI = 0.54; 4.99), indicating no significant association with suicide ideation for adolescents who used any psychotropic recreational drug at least once in their lives compared to those who never used it (Figure 2). A high level of heterogeneity was also observed (I^2^ = 98.6%). This is justified by a study by Yip et al.^42^, which indicated that drug use was a protective factor (OR < 1.00).

**Figure 3:**
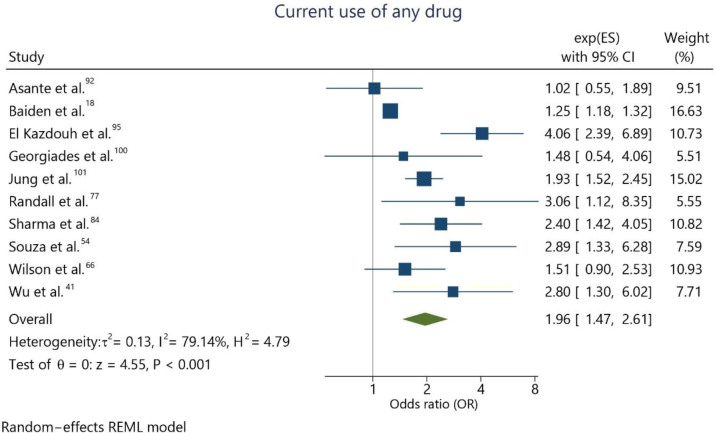
Meta-analysis assessing the recurrent use of any illicit drug and suicidal ideation.

**Figure 4:**
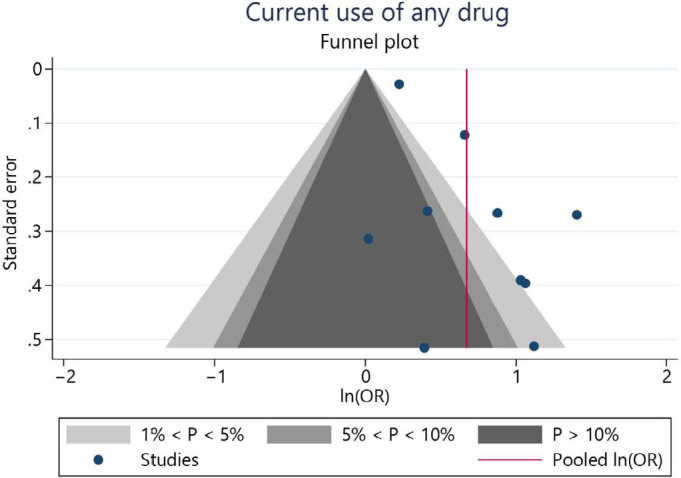
Funnel plot indicating a potential risk of publication bias in the recurrent use of any illicit drugs analysis.

Regarding the recurrent use of psychotropic recreational drugs, ten studies^18,41,54,66,77,84,92,95,100,101^ analyzed its association with suicidal ideation. The combined effect estimation for suicidal ideation was OR 1.96 (95%CI = 1.47; 2.61) for adolescents who reported currently using psychotropic recreational drugs compared to non-users (Figure 3). Heterogeneity was high (I^2^ = 79.1%). The funnel plot indicates the potential risk of publication bias (Figure 4).

**Figure 5:**
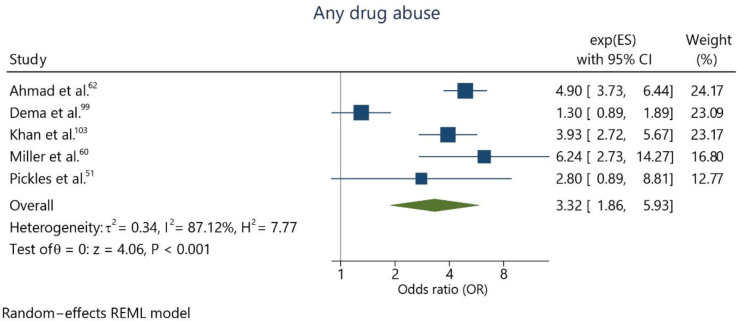
Meta-analysis assessing the abuse of any illicit drug and suicidal ideation.

Five studies^51,60,62,99,103^ analyzed the relationship between the use or abuse of any psychotropic recreational drugs and suicide ideation. The combined effect estimation for adolescents who abused drugs compared to those who did not was OR 3.32 (95%CI = 1.86; 5.93) (Figure 5), with considerably high heterogeneity (I² = 87.1%).

### Cannabis use

**Figure 6:**
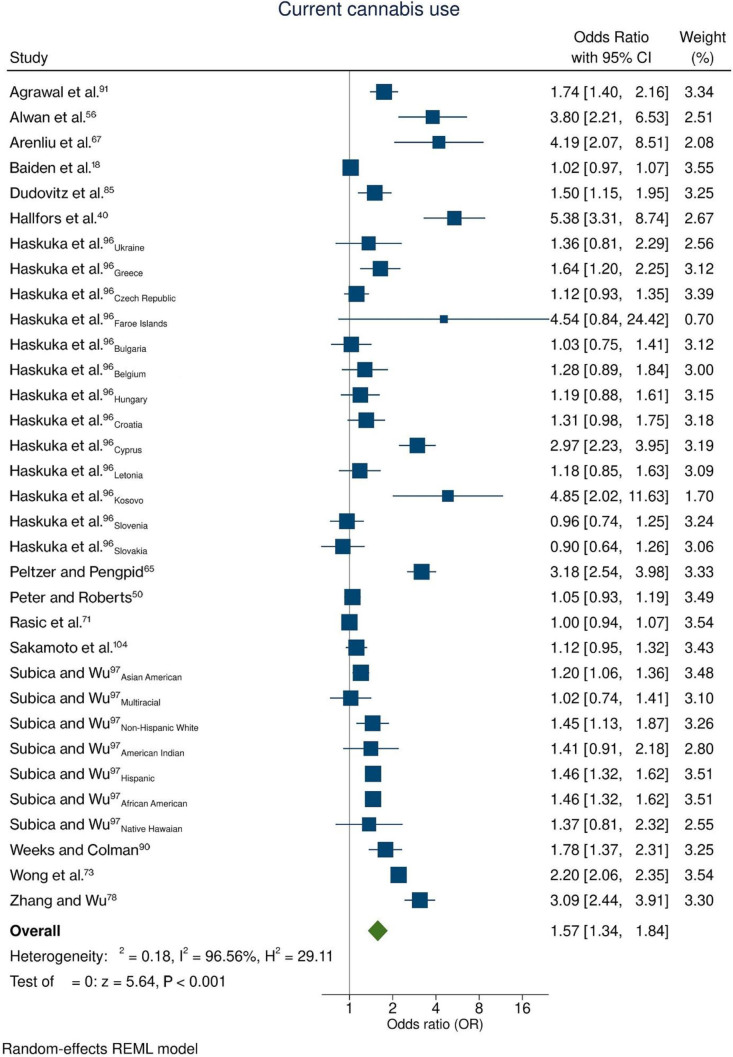
Meta-analysis assessing the recurrent use of cannabis and suicidal ideation.

**Figure 7:**
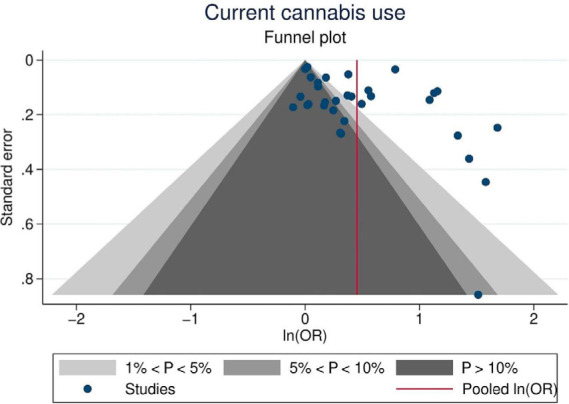
Funnel plot indicating a potential risk of publication bias in the recurrent use of cannabis analysis.

Fifteen studies^17,18,40,50,56,67,71,73,78,85,90,91,96,97,104^ provided sufficient data on the likelihood of suicide ideation related to cannabis use. Additionally, these studies provided 33 databases that were included in the meta-analysis. Overall, adolescents who reported currently using cannabis were 1.57 times more likely (95%CI = 1.34; 1.84) to present suicide ideation than non-users (Figure 6). The heterogeneity was considered high (I^2^ = 96.6%). The funnel plot indicated a high probability of publication bias (Figure 7).

### Cocaine use

**Figure 8:**
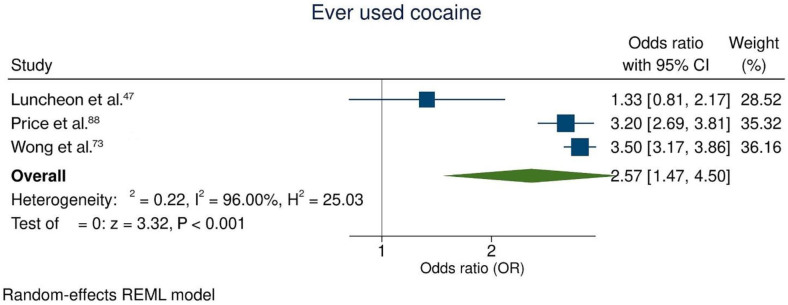
Meta-analysis assessing the use of cocaine and suicide ideation.

Only three studies^47,73,88^ analyzed the likelihood of suicide ideation relative to cocaine use. Suicide ideation was 2.57 times more likely (95%CI = 1.47; 4.50; I² = 96.0%) for adolescents who used cocaine at some point in their lives than for those who never used the drug (Figure 8).

### Certainty of evidence

All outcomes indicated a very low certainty of evidence (Table 5), downgraded due to inconsistency, imprecision, or potential publication bias.

**Table 5:** Grading of Recommendations Assessment, Development, and Evaluation (GRADE) Summary of Findings Table for the Outcomes of the Systematic Review

Certainty assessment							Effect	Certainty
Number of studies	Study design	Risk of bias	Inconsistency	Indirectness	Imprecision	Other considerations	Relative (95% CI)	
**Current use of any illicit drug vs. no use**								
10	observational studies	not serious	serious^a^	not serious	not serious	Publication bias detected	OR 1.96 (1.47 to 2.61)	⨁◯◯◯VERY LOW
**Ever used any illicit drug vs. no use**								
4	observational studies	not serious	serious^a^	not serious	very serious^b^	none^d^	OR 1.65 (0.54 to 4.99)	⨁◯◯◯VERY LOW
**Any illicit drug abuse vs. no use**								
5	observational studies	not serious	serious^a^	not serious	serious^c^	none^d^	OR 3.32 (1.86 to 5.93)	⨁◯◯◯VERY LOW
**Current cannabis use vs. no use**								
15	observational studies	not serious	serious^a^	not serious	not serious	Publication bias detected	OR 1.57(1.34 to 1.84)	⨁◯◯◯VERY LOW
**Ever used cocaine vs. no use**								
3	observational studies	not serious	serious^a^	not serious	serious^c^	none^d^	OR 2.57(1.47 to 4.50)	⨁◯◯◯VERY LOW

CI = confidence interval; OR = odds ratio,

^a^ high unexplained statistical heterogeneity (I^2^ ＞ 50%) and/or no overlapping of effect estimates – Rated down by one level;

^b^ confidence interval suggests trivial no association in one extreme and strong association in another – Rated down by two levels;

^c^ confidence interval suggests trivial association in one extreme and strong association in another – Rated down by one level;

^d^ publication bias was not assessed due to the low number of studies.

**GRADE Working Group grades of evidence**

**High certainty:** Very confident that the true effect is close to the effect estimate.

**Moderate certainty:** Moderately confident in the effect estimate: The true effect is likely close to the effect estimate but may be substantially different.

**Low certainty:** The confidence in the effect estimate is limited: The true effect may differ substantially from the effect estimate.

**Very low certainty:** Very little confidence in the effect estimate: The true effect may differ substantially from the effect estimate.

## DISCUSSION

This systematic review and meta-analysis aimed to investigate the association between suicide ideation and psychotropic recreational drug use among adolescents. The findings indicate that drug use is associated with higher odds of suicide ideation in adolescents.

Suicide ideation among adolescents is a prevalent social problem globally^20,105^, with varying prevalence rates observed across studies conducted in different countries. For instance, a Korean study conducted between 2018 and 2019 found that approximately 4.0% of adolescents reported having contemplated suicide, with about 3.0% attempting suicide, and substance use was identified as a contributing risk factor.^105^ Similarly, in China, the prevalence of suicide ideation among the young population was reported at 26.4%, with higher rates observed among those with a smoking habit.^106^Canadian adolescents experienced a surge in suicidal ideation, reaching 44% during the coronavirus disease 2019 (COVID-19) pandemic.^107^ A multicenter study comprising 77 countries indicated a weighted prevalence of 18% for suicide ideation among adolescents.^108^ Thus, suicide ideation can be considered a frequent event among youth, warranting urgent attention, especially considering that adolescents with suicide ideation are reportedly 12 times more likely to attempt suicide by the age of 30.^109^

While some studies suggest a higher prevalence among female adolescents,^29,62,87,110^ others demonstrated a higher prevalence among male adolescents.^67,74,95,111,112^ Studies that did not verify differences between the sexes were also identified.^94,102^ This lack of consensus may arise from cultural factors^113^ or may signify the absence of inherent differences between the sexes.

Overall, the analyses conducted in this review indicated that adolescents who engage in psychotropic recreational drug use are more susceptible to experiencing suicide ideation. Furthermore, the meta-analysis results suggest that the higher the frequency of substance use, the higher the likelihood of suicidal ideation. This correlation can be attributed to various factors associated with adolescent drug use, including depression, stress throughout life, familial conflicts, and exposure to bullying and moral abuse in school settings, all of which act as risk factors for suicide ideation. However, two studies included in the meta-analysis did not identify statistically significant differences in suicide ideation between adolescents who did or did not use psychotropic recreational drugs .^66,92^ Notably, these two studies were performed in African countries and indicated the possibility of data underreporting, considering the low number of positive answers to psychotropic recreational drug use.

Additionally, the meta-analysis revealed that cannabis was linked to an increased likelihood of developing suicidal ideation among adolescents. Chronic cannabis use during adolescence has been associated with the onset of mental disorders,^114,^ and including psychosis, schizophrenia, schizoaffective disorders, and a high risk of mood disorders.^115^ Cannabis use before the age of 15 years and frequent recent use further increase the risk of suicidal ideation.^114^ Moreover, cannabis use was associated with a higher prevalence of suicidal ideation and deliberate self-harm among Canadian adolescents evaluated during the COVID-19 pandemic.^107^

The meta-analysis results potentially indicate that cocaine use is associated with suicide ideation. However, the high heterogeneity and low number of studies assessing this outcome suggest that further studies are required to confirm this association. Exposure to cocaine starting in adolescence increases vulnerability to developing drug dependence and decreases individuals’ likelihood of seeking treatment.^116^ Moreover, cannabis and cocaine use are associated with a higher prevalence of the first psychotic episode and the incidence of the first prodromal or psychotic symptom earlier than individuals who only use tobacco.^117^

The analysis could not fully explore the relationship between suicide ideation and other drugs, such as LSD, heroin, or methamphetamine, due to the small number of studies specifying the type of substance used. Further studies using detailed questionnaires to differentiate between the types of drugs used are warranted.

In the public health context, developing robust surveillance systems for suicide prevention is imperative.^118^ Various policies, including firearm access restrictions, awareness of communication media on the importance of addressing the topic correctly, identification of people at risk, ^13^ and the provision of psychotherapeutic, pharmacological, or neuromodulatory treatment, and training of health professionals to identify people at risk for suicide and offer proper care, are essential for effective suicide prevention efforts.^119^

Programs for drug use prevention targeting adolescent drug use should incorporate discussions on bullying (both practicing and being a victim)^9^ and strategies for addressing associated problems, such as interpersonal conflicts, anxiety, loneliness, an intimidating environment, a lack of support from parents and close friends, sedentary behavior,^108^ and drug use, as evidenced in the present systematic review. A systematic review and meta-analysis concluded that the development of interpersonal skills, emotional regulation, and alcohol and drug education significantly impacted mental health^120^. Therefore, this intervention should start in childhood to increase an individual’s ability to deal with adverse and unwanted situations.

School-based programs, like the ‘Unplugged’ program, comprising 12 one-hour interactive sessions by trained teachers to address social and personal skills and attitudes towards drugs, were implemented in Europe (Italy, Greece, Spain, Austria, Belgium, Germany, and Sweden) in 2003, presenting promising results in decreasing tobacco and cannabis use among adolescents.^121^ However, adaptations of such programs, like the “#tamojunto’ project in Brazilian schools, did not present a difference in substance use between the experimental and the control group.^122^

A potential limitation of this study could be the presence of social desirability bias, in which participants may have been inclined to provide socially acceptable answers, potentially distorting the accuracy of their answers to conform to societal norms.^123^ Consequently, there may have been an underreporting of adolescents acknowledging drug use. Moreover, most the majority of eligible studies did not specify whether adolescents were screened for substance use, potentially excluding individuals who had previously used psychoactive substances from the analysis. Another factor that may undermine the generalizability of our results is the predominance of the eligible studies conducted in high-income countries. Given the multifactorial origins of suicidal ideation and drug use, further research is needed to comprehensively elucidate these factors, particularly within diverse socioeconomic contexts^124^.

Despite these limitations, our results are relevant because this is the first systematic review investigating the relationship between psychotropic recreational drug use and suicide ideation among adolescents. Additionally, we should emphasize the large number of eligible studies,^78^ including gray literature, and the assessment of results from studies performed worldwide. Further studies in developing countries are suggested, especially in Africa and Latin America, for better analysis and extrapolation of results, and more specific details regarding the use and specific types of drugs.

## CONCLUSIONS

This systematic review confirmed the association between psychotropic recreational drug use and suicide ideation among adolescents, irrespective of their current or previous use, abuse, or specific type of substance used. Adolescents who currently use cannabis or cocaine exhibit a higher likelihood of experiencing suicide ideation than those who do not engage in psychotropic recreational drug use. Policymakers and health professionals should be aware that suicidal behavior is multifaceted and not solely attributed to substance use or abuse. Furthermore, both suicide ideation and psychotropic recreational drug use share common predictors, underscoring the interwoven nature of these phenomena.

## References

[1] Valle LELR, Mattos MJVM (2011). Adolescência: as contradições da idade. Rev Psicopedagog..

[2] Carbonário FA (2018). Neurociência do abuso de drogas na adolescência. Mental..

[3] Guo L, Wang W, Du X (2021). Associations of substance use behaviors with suicidal ideation and suicide attempts among US and Chinese adolescents. Front Psychiatry..

[4] United Nations Office on Drugs and Crime (UNODC) (2018). International Narcotics Control Board: Report of the International Narcotics Control Board for 2017.

[5] Basith SA, Nakaska MM, Sejdiu A (2021). Substance use disorders (SUD) and suicidal behaviors in adolescents: insights from cross-sectional inpatient study. Cureus..

[6] Nowak K, Ratajczak-Wrona W, Górska M, Jabłońska E (2018). Parabens and their effects on the endocrine system. Mol Cell Endocrinol..

[7] Silva DMRD, Costa DT, Rocha GSDA (2021). Association between family dynamics and use of alcohol, tobacco, and other drugs by adolescents. Rev Bras Enferm..

[8] Mehanović E, Vigna-Taglianti F, Faggiano F, Galanti MR, EU-Dap Study Group (2022). Does parental permissiveness toward cigarette smoking and alcohol use influence illicit drug use among adolescents? A longitudinal study in seven European countries. Soc Psychiatry Psychiatr Epidemiol..

[9] Shawki B, Al-Hadithi T, Shabila N (2021). Association of bullying behaviour with smoking, alcohol use and drug use among school students in Erbil City, Iraq. Eastern Med Health J..

[10] Aschengrau A, Grippo A, Winter MR (2021). Influence of Family and Community Socioeconomic Status on the Risk of Adolescent Drug Use. Subst Use Misuse..

[11] United Nations Office on Drugs and Crime (UNODC) (2020). United Nations Convention against Transnational Organized Crime and Palermo Protocols. Office on Drugs and Crime.

[12] World Health Organization (2018). Informações sobre Prevenção ao Suicídio (SUPRE).

[13] World Health Organization (2019). Preventing suicide: a resource for pesticide registrars and regulators.

[14] Castro M, Cunha SS, Souza DP (2011). Violence behavior and factors associated among students of Central-West Brazil. Rev Saúde Pública..

[15] World Health Organization (2017). Dados de suicídio.

[16] Stark L, Seff I, Yu G (2022). Correlates of Suicide Ideation and Resilience Among Native- and Foreign-Born Adolescents in the United States. J Adolesc Health..

[17] Peltzer K, Pengpid S (2015). Early Substance Use Initiation and Suicide Ideation and Attempts among School-Aged Adolescents in Four Pacific Island Countries in Oceania. Int J Environment Res Public Health..

[18] Baiden P, LaBrenz CA, Asiedua-Baiden G, Muehlenkamp JJ (2020). Examining the intersection of race/ethnicity and sexual orientation on suicidal ideation and suicide attempt among adolescents: Findings from the 2017 Youth Risk Behavior Survey. J Psych Res..

[19] Gijzen M, Rasing S, Creemers D (2021). Suicide ideation as a symptom of adolescent depression. a network analysis. J Affect Disord..

[20] Correa Díaz EP, Jácome Sánchez EC, Martínez BA (2015). Suicide in adolescents with depression: the need for early diagnosis. Clinic Case Reports..

[21] Breet E, Goldstone D, Bantjes J (2018). Substance use and suicidal ideation and behaviour in low- and middle-income countries: a systematic review. BMC Public Health..

[22] Moher D, Shamseer L, Clarke M (2015). Preferred reporting items for systematic review and meta-analysis protocols (PRISMA-P) 2015 statement. Syst Rev..

[23] Page MJ, Moher D, Bossuyt PM, Boutron I, Hoffmann TC, Mulrow CD (2021). PRISMA 2020 explanation and elaboration: updated guidance and exemplars for reporting systematic reviews. BMJ.

[24] Aromataris E, Munn Z (2020). JBI Manual for Evidence Synthesis.

[25] Moola S, Munn Z, Tufanaru C, Aromataris E, Munn Z (2020). JBI Manual for Evidence Synthesis..

[26] Sampaio MS, Vieira WDA, Bernardino IDM (2019). Chronic obstructive pulmonary disease as a risk factor for suicide: a systematic review and meta-analysis. Resp Med..

[27] Page MJ, Higgins JPT, Sterne JAC, Higgins J, Thomas J (2021). Cochrane Handbook for Systematic Reviews of Interventions..

[28] Guyatt G, Oxman AD, Akl EA (2011). GRADE guidelines: 1. Introduction-GRADE evidence profiles and summary of findings tables. J Clin Epidemiol..

[29] Kandel DB, Raveis VH, Davies M (1991). Suicidal ideation in adolescence: Depression, substance use, and other risk factors. J Youth Adol..

[30] Felts WM, Chernier T, Barnes R (1992). Drug use and suicide ideation and behavior among North Carolina public school students. Am J Public Health..

[31] Garrison CZ, McKeown RE, Valois RF, Vincent ML (1993). Aggression, substance use, sand suicidal behaviors in high school students. Am J Public Health..

[32] Vega WA, Gil A, Warheit G, Apospori E, Zimmerman R (1993). The relationship of drug use to suicide ideation and attempts among African American, Hispanic, and white non-Hispanic male adolescents. Suicide Life-threat Behav..

[33] Madianos MG, Gefou-Madianou D, Stefanis CN (1994). Symptoms of depression, suicidal behaviour and use of substances in Greece: a nationwide general population survey. Acta Psyc Scandinav..

[34] Burge V, Felts M, Chenier T, Parrillo AV (1995). Drug use, sexual activity, and suicidal behavior in U.S. high school students. J School Health..

[35] López L, Elsa K, Medina M (1995). La relación entre la ideación suicida y el abuso de sustancias tóxicas: resultado de una encuesta en la población estudiantil. Salud ment..

[36] Windle RC, Windle M (1997). An investigation of adolescents’ substance use behaviors, depressed affect, and suicidal behaviors. J Child Psychol Psychiatry..

[37] Simon TR, Crosby AE (2000). Suicide planning among high school students who report attempting suicide. Suicide Life Threat Behav..

[38] Perkins DF, Glen H (2002). An ecological risk-factor examination of suicide ideation and behavior of adolescents. J Adoles Research..

[39] Vermeiren R, Schwab-Stone M, Ruchkin VV (2003). Suicidal behavior and violence in male adolescents: a school-based study. J Am Acad Child Adolesc Psychiatry..

[40] Hallfors DD, Waller MW, Ford CA (2004). Adolescent depression and suicide risk: association with sex and drug behavior. Am J Prev Med..

[41] Wu P, Hoven CW, Liu X (2004). Substance use, suicidal ideation and attempts in children and adolescents. Suicide Life Threat Behav..

[42] Yip PS, Liu KY, Lam TH (2004). Suicidality among high school students in Hong Kong, SAR. Suicide Life Threat Behav..

[43] Spremo M, Loga S (2005). The relationship between suicidal thoughts and psychoactive substances. Bosn J Basic Med Sci..

[44] Ulusoy MD, Demir NO (2005). Suicidal ideation in Turkish adolescents. Soc Behav Pers..

[45] Dunn MS, Goodrow B, Givens C, Austin S (2008). Substance use behavior and suicide indicators among rural middle school students. J School Health..

[46] Chabrol H, Chauchard E, Girabet J (2008). Cannabis use and suicidal behaviours in high-school students. Addict Behav..

[47] Luncheon C, Bae S, Gonzalez A, Lurie S, Singh KP (2008). Hispanic female adolescents’ use of illicit drugs and the risk of suicidal thoughts. Am J Health Behav..

[48] Peltzer K, Kleintjes S, Van Wyk B, Thompson EA, Mashego T (2008). Correlates of suicide risk among secondary school students in Cape Town. Soc Behav Pers..

[49] Epstein JA, Spirito A (2009). Risk factors for suicidality among a nationally representative sample of high school students. Suicide Life Threat Behav..

[50] Peter T, Roberts LW (2010). ‘Bad’ boys and ‘sad’ girls? Examining internalizing and externalizing effects on parasuicides among youth. J Youth Adol..

[51] Pickles A, Aglan A, Collishaw S (2010). Predictors of suicidality across the life span: the Isle of Wight study. Psych Med..

[52] Florenzano R, Cáceres E, Valdés M (2010). Comparación de frecuencia de conductas de riesgo, problemas juveniles y estilos de crianza, en estudiantes adolescentes de tres ciudades chilenas. Cuad Méd Soc..

[53] Page RM, West JH, Hall PC (2011). Psychosocial distress and suicide ideation in Chinese and Philippine adolescents. Asia Pac J Public Health..

[54] Souza LD, Ores L, Oliveira GT (2010). Ideação suicida na adolescência: prevalência e fatores associados. J Bras Psiq..

[55] Wolitzky-Taylor KB, Ruggiero KJ, McCart MR (2010). Has adolescent suicidality decreased in the United States? Data from two national samples of adolescents interviewed in 1995 and 2005. J Clin Child Adolesc Psychol..

[56] Alwan H, Viswanathan B, Rousson V, Paccaud F, Bovet P (2011). Association between substance use and psychosocial characteristics among adolescents of the Seychelles. BMC Ped..

[57] Carvalho PD, Barros MV, Lima RA, Santos CM, Mélo EN (2011). Condutas de risco à saúde e indicadores de estresse psicossocial em adolescentes estudantes do Ensino Médio. Cad Saúde Pública..

[58] Eaton DK, Foti K, Brener ND (2011). Associations between risk behaviors and suicidal ideation and suicide attempts: do racial/ethnic variations in associations account for increased risk of suicidal behaviors among Hispanic/Latina 9th- to 12th-grade female students?. Arch Suicide Res..

[59] Kim J, Fan B, Liu X, Kerner N, Wu P (2011). Ecstasy use and suicidal behavior among adolescents: findings from a national survey. Suicide Life Threat Behav..

[60] Miller M, Borges G, Orozco R (2011). Exposure to alcohol, drugs and tobacco and the risk of subsequent suicidality: findings from the Mexican Adolescent Mental Health Survey. Drug Alcohol Depend..

[61] Swahn MH, Bossarte RM, Choquet M (2012). Early substance use initiation and suicide ideation and attempts among students in France and the United States. Int J Public Health..

[62] Ahmad N, Cheong SM, Ibrahim N, Rosman A (2012). Suicidal ideation among Malaysian adolescents. Asia Pac J Public Health..

[63] Bakken NW, Gunter WD (2012). Self-cutting and suicidal ideation among adolescents: Gender differences in the causes and correlates of self-injury. Deviant Behav..

[64] Kokkevi A, Rotsika V, Arapaki A, Richardson C (2012). Adolescents’ self-reported suicide attempts, self-harm thoughts and their correlates across 17 European countries. J Child Psychol Psychiatry..

[65] Peltzer K, Pengpid S (2012). Suicidal ideation and associated factors among school-going adolescents in Thailand. Int J Environ Res Public Health..

[66] Wilson ML, Dunlavy AC, Viswanathan B, Bovet P (2012). Suicidal expression among school-attending adolescents in a middle-income sub-Saharan country. Int J Environ Res Public Health..

[67] Arenliu A, Kaltrina K, Mytaher H, Teuta H, Ercan C (2014). Drug use and reported suicide ideation and attempt among Kosovar adolescents. J Substance Use..

[68] Consoli A, Peyre H, Speranza M, Hassler C, Falissard B, Touchette E (2013). Suicidal behaviors in depressed adolescents: role of perceived relationships in the family. Child Adolesc Psychiatry Ment Health..

[69] Delfabbro PH, Winefield HR, Winefield AH (2013). Life-time and current suicide-ideation in Australian secondary school students: Socio-demographic, health and psychological predictors. J Affect Disord..

[70] Govender K, Naicker SN, Meyer-Weitz A (2013). Associations between perceptions of school connectedness and adolescent health risk behaviors in South African high school learners. J School Health..

[71] Rasic D, Weerasinghe S, Asbridge M, Langille DB (2013). Longitudinal associations of cannabis and illicit drug use with depression, suicidal ideation and suicidal attempts among Nova Scotia high school students. Drug Alcohol Depend..

[72] Shilubane HN, Ruiter RA, van den Borne B (2013). Suicide and related health risk behaviours among school learners in South Africa: results from the 2002 and 2008 national youth risk behaviour surveys. BMC Public Health..

[73] Wong JP (2013). Presentation to Global Health & Health Equity Forum..

[74] Van Ours JC, Williams J, Fergusson D, Horwood LJ (2013). Cannabis use and suicidal ideation. J Health Economics..

[75] Chabrol H, Melioli T, Goutaudier N (2014). Cannabis use and suicidal ideations in high-school students. Addictive Behav..

[76] Lowry R, Crosby AE, Brener ND, Kann L (2014). Suicidal thoughts and attempts among u.s. High school students: trends and associated health-risk behaviors, 1991-2011. J Adolesc Health..

[77] Randall JR, Doku D, Wilson ML, Peltzer K (2014). Suicidal behaviour and related risk factors among school-aged youth in the Republic of Benin. PloS one..

[78] Zhang X, Wu LT (2014). Suicidal ideation and substance use among adolescents and young adults: a bidirectional relation?. Drug Alcohol Depend..

[79] Delfabbro HP, Malvaso C, Winefield HA, Winefield RH (2015). Socio-demographic, health, and psychological correlates of suicidality severity in Australian adolescents. Aust J Psychol..

[80] Dunlavy AC, Aquah EO, Wilson ML (2015). Suicidal ideation among school-attending adolescents in Dar es Salaam, Tanzania. Tanzan J Health Res..

[81] Gart R, Kelly S (2015). How Illegal Drug Use, Alcohol Use, Tobacco Use, and Depressive Symptoms Affect Adolescent Suicidal Ideation: A Secondary Analysis of the 2011 Youth Risk Behavior Survey. Issues Ment Health Nurs..

[82] Lee GY, Choi YJ (2015). Association of school, family, and mental health characteristics with suicidal ideation among Korean adolescents. Res Nurs Health..

[83] Sampasa-Kanyinga H, Dupuis LC, Ray R (2017). Prevalence and correlates of suicidal ideation and attempts among children and adolescents. Int J Adolesc Med Health..

[84] Sharma B, Nam EW, Kim HY, Kim JK (2015). Factors Associated with Suicidal Ideation and Suicide Attempt among School-Going Urban Adolescents in Peru. Int J Environ Res Public Health..

[85] Dudovitz RN, McCoy K, Chung PJ (2015). At-school substance use as a marker for serious health risks. Academic Ped..

[86] Ammerman BA, Steinberg L, McCloskey MS (2018). Risk-Taking Behavior and Suicidality: The Unique Role of Adolescent Drug Use. J Clin Child Adolesc Psychol..

[87] DeCamp W, Bakken NW (2016). Self-injury, suicide ideation, and sexual orientation: differences in causes and correlates among high school students. J Inj Violence Res..

[88] Price JH, Khubchandani J (2017). Latina Adolescents Health Risk Behaviors and Suicidal Ideation and Suicide Attempts: Results from the National Youth Risk Behavior Survey 2001-2013. J Immigr Minor Health..

[89] Kim EM, Kim H, Park E (2020). How are depression and suicidal ideation associated with multiple health risk behaviours among adolescents? A secondary data analysis using the 2016 Korea Youth Risk Behavior Web-based Survey. J Psychiatr Ment Health Nurs..

[90] Weeks M, Colman I (2017). Predictors of Suicidal Behaviors in Canadian Adolescents with No Recent History of Depression. Arch Suic Research..

[91] Agrawal A, Tillman R, Grucza RA (2017). Reciprocal relationships between substance use and disorders and suicidal ideation and suicide attempts in the Collaborative Study of the Genetics of Alcoholism. J Affect Disord..

[92] Asante KO, Kugbey N, Osafo J, Quarshie EN, Sarfo JO (2017). The prevalence and correlates of suicidal behaviours (ideation, plan and attempt) among adolescents in senior high schools in Ghana. SSM Popul Health..

[93] Janssen E, Spilka S, Beck F (2017). Suicide, santé mentale et usages de substances psychoactives chez les adolescents français en 2014. Rev Epidemiol Sante Publique..

[94] Wang PW, Yen CF (2017). Adolescent substance use behavior and suicidal behavior for boys and girls: a cross-sectional study by latent analysis approach. BMC Psych..

[95] El Kazdouh H, El-Ammari A, Bouftini S, El Fakir S, El Achhab Y (2019). Potential risk and protective factors of substance use among school adolescents in Morocco: A cross-sectional study. J Subst Use..

[96] Haskuka M, Aliriza A, Kaltrina K (2018). The relationship between substance use and suicidal behaviour among adolescents in selected European countries: A test of normalisation theory. Drugs..

[97] Subica AM, Wu LT (2018). Substance Use and Suicide in Pacific Islander, American Indian, and Multiracial Youth. Am J Prev Med..

[98] Chadi N, Li G, Cerda N, Weitzman ER (2019). Depressive Symptoms and Suicidality in Adolescents Using e-Cigarettes and Marijuana: A Secondary Data Analysis From the Youth Risk Behavior Survey. J Addict Med..

[99] Dema T, Tripathy JP, Thinley S (2019). Suicidal ideation and attempt among school going adolescents in Bhutan - a secondary analysis of a global school-based student health survey in Bhutan 2016. BMC Public Health..

[100] Georgiades K, Boylan K, Duncan L (2019). Prevalence and Correlates of Youth Suicidal Ideation and Attempts: Evidence from the 2014 Ontario Child Health Study. Canadian journal of psychiatry. Can J Psychiatry..

[101] Jung JS, Park SJ, Kim EY (2019). Prediction models for high risk of suicide in Korean adolescents using machine learning techniques. PloS one..

[102] Greene N, Tomedi L, Reno J, Green D (2020). The Role of Substance Use and Resiliency Factors on Suicidal Ideation among Middle School Students. J Sch Health..

[103] Khan M, Rahman MM, Islam MR (2020). Suicidal behavior among school-going adolescents in Bangladesh: findings of the global school-based student health survey. Soc Psychiatry Psychiatr Epidemiol..

[104] Sakamoto JL, Shibanuma A, Jimba M (2020). Depressed mood, suicidal behaviors, and health risk behaviors among youths in the Commonwealth of the Northern Mariana Islands: the 2017 CNMI Youth Risk Behavior Survey. BMC Public Health..

[105] Lee JW, Kim BJ, Lee CS (2021). Association Between Suicide and Drinking Habits in Adolescents. J Child Adolesc Psych..

[106] Hu J, Song X, Li D (2021). Interaction of smoking and being bullied on suicidal behaviors: a school-based cross-sectional survey in China. Environ Health Prev Med..

[107] Turner BJ, Robillard CL, Ames ME, Craig SG (2022). Prevalence and Correlates of Suicidal Ideation and Deliberate Self-harm in Canadian Adolescents During the Coronavirus Disease 2019 Pandemic. Canadian journal of psychiatry. Can J Psychiatry..

[108] Mahumud RA, Dawson AJ, Chen W (2022). The risk and protective factors for suicidal burden among 251,763 school-based adolescents in 77 low- and middle-income to high-income countries: assessing global, regional, and national variations. Psych Med..

[109] Cha CB, Franz PJ, Guzmán M (2018). Annual Research Review: Suicide among youth - epidemiology, (potential) etiology, and treatment. J Child Psychol Psychiatry..

[110] Gmitrowicz A, Szymczak W, Kotlicka-Antczak M (2003). Rabe-Jabłońska J. Suicidal ideation and suicide attempt in Polish adolescents: is it a suicidal process?. Int J Adolesc Med Health..

[111] Torikka A, Kaltiala-Heino R, Marttunen M (2002). Drinking, other substance use and suicidal ideation in middle adolescence: a population study. J Substance Use..

[112] Franić T, Dodig G, Kardum G (2011). Early adolescence and suicidal ideations in Croatia: sociodemographic, behavioral, and psychometric correlates. Crisis..

[113] Owusu A (2008). Ghana Country report on the Global School-based Health Survey (GSHS).

[114] Borges G, Bagge CL, Orozco R (2016). A literature review and meta-analyses of cannabis use and suicidality. J Affect Disord..

[115] Hamidullah S, Thorpe H, Frie JA, Mccurdy RD, Khokhar JY (2020). Adolescent Substance Use and the Brain: Behavioral, Cognitive and Neuroimaging Correlates. Frontiers in Human Neuroscience..

[116] DePoy LM, Zimmermann KS, Marvar PJ, Gourley SL (2017). Induction and Blockade of Adolescent Cocaine-Induced Habits. Biol Psych..

[117] González-Blanco L, García-Portilla MP, Gutiérrez M (2021). Impact of previous tobacco use with or without cannabis on first psychotic experiences in patients with first-episode psychosis. Schizophr Research..

[118] Ertl A, Sheats KJ, Petrosky E (2019). Surveillance for Violent Deaths - National Violent Death Reporting System, 32 States, 2016. Surveillance Summaries..

[119] Naguy A, Elbadry H, Salem H (2020). Suicide: A Précis!. J Family Med Prim Care..

[120] Skeen S, Laurenzi CA, Gordon SL (2019). Adolescent Mental Health Program Components and Behavior Risk Reduction: A Meta-analysis. Pediatrics..

[121] Vigna-Taglianti FD, Galanti MR, Burkhart G (2014). “Unplugged,” a European school-based program for substance use prevention among adolescents: overview of results from the EU-Dap trial. New Dir Youth Dev..

[122] Sanchez ZM, Valente JY, Sanudo A, Pereira APD, Cruz JI, Schneider D, Andreoni S (2017). The #Tamojunto Drug Prevention Program in Brazilian Schools: a Randomized Controlled Trial. Prev Sci..

[123] Stuart GS, Grimes DA (2009). Social desirability bias in family planning studies: a neglected problem. Contraception..

[124] Czyz EK, Horwitz AG, Arango A, King CA (2019). Short-term change and prediction of suicidal ideation among adolescents: a daily diary study following psychiatric hospitalization. J Child Psychol Psychiatry..

